# Cell-Based Therapies for Traumatic Brain Injury: Therapeutic Treatments and Clinical Trials

**DOI:** 10.3390/biomedicines9060669

**Published:** 2021-06-10

**Authors:** Celia Bonilla, Mercedes Zurita

**Affiliations:** 1 Cell Therapy Unit, Puerta de Hierro Hospital, 28222 Majadahonda, Madrid, Spain; 2 Cell Therapy Unit Responsable, Puerta de Hierro Hospital, 28222 Majadahonda, Madrid, Spain; mercedes.zurita@salud.madrid.org

**Keywords:** traumatic brain injury, stem cells, clinical studies, clinical trials, stem cell transplantation

## Abstract

Traumatic brain injury (TBI) represents physical damage to the brain tissue that induces transitory or permanent neurological disabilities. TBI contributes to 50% of all trauma deaths, with many enduring long-term consequences and significant medical and rehabilitation costs. There is currently no therapy to reverse the effects associated with TBI. An increasing amount of research has been undertaken regarding the use of different stem cells (SCs) to treat the consequences of brain damage. Neural stem cells (NSCs) (adult and embryonic) and mesenchymal stromal cells (MSCs) have shown efficacy in pre-clinical models of TBI and in their introduction to clinical research. The purpose of this review is to provide an overview of TBI and the state of clinical trials aimed at evaluating the use of stem cell-based therapies in TBI. The primary aim of these studies is to investigate the safety and efficacy of the use of SCs to treat this disease. Although an increasing number of studies are being carried out, few results are currently available. In addition, we present our research regarding the use of cell therapy in TBI. There is still a significant lack of understanding regarding the cell therapy mechanisms for the treatment of TBI. Thus, future studies are needed to evaluate the feasibility of the transplantation of SCs in TBI.

## 1. Introduction

TBI is a leading global cause of mortality and morbidity [1,2] and the main cause of death in young people living in industrialized countries [3]. TBI is mainly caused by an external mechanical force causing brain trauma. This trauma can lead to temporary or permanent dysfunctions that induce cognitive, physical, and emotional disturbances [4,5]. Long-term disability is linked to the severity of the initial brain injury, the diffuse axonal injury (DAI), and the posterior neuro-rehabilitation [6,7,8].

TBI and the ensuing neuroinflammation, in addition to causing motor and cognitive deficits, may persist long after the initial injury [9]. Furthermore, long-term neuroinflammation has been related to increased risk of neurodegenerative disorders and neurobehavioral deficits, including Alzheimer’s disease, Parkinson’s disease, amyotrophic lateral sclerosis (ALS), and chronic traumatic encephalopathy [10]. TBI has also been linked with other mental health disorders, such as depression, anxiety or psychotic disorders, and cognitive disorders related to executive functioning and aggression [11].

Thus, although TBI is a significant public health problem, unfortunately there is no an effective therapy that has proved efficacious in its treatment. Numerous pharmacological treatments of TBI exist to reduce neurological damage [12], but none is sufficiently effective to reverse the resulting neurological deficit. Therapies used after TBI are limited largely to rehabilitation [13,14] and some palliative drugs [11]. Neuro-rehabilitation may ameliorate some deficits, but ultimately fails to provide neuro-restorative outcomes. About half of people suffering TBI do not return to their previous work after 1 year, and ~28% never returned to work of any kind [15]. Patients who survive TBI can develop serious deficits in sensorial and motor function [16], and the possibility of treating the devastating consequences of TBI is currently one of the main challenges in neurobiology.

In recent decades, researchers have focused on the use of SCs as a potential therapeutic option for the treatment of TBI. SCs can both provide a neuroprotection role and participate in tissue reconstruction [17], with the potential to not only modulate systemic inflammation, but also provide multiple neurorestorative benefits by simultaneous promotion of neurogenesis, angiogenesis, and neuroprotection at the site of injury [18]. Several cell types have been tested for post-TBI therapy, including NSCs (adult and embryonic), induced pluripotent SCs (iPSCs), and MSCs, such as adipose tissue-derived MSCs (AD-MSCs), bone marrow-derived MSCs (BM-MSCs), and umbilical cord-derived MSCs (UC-MSCs) [19]. All of these cell types have demonstrated the ability to improve neurological outcomes and recovery in preclinical trials investigating TBI models.

This review aims to provide an overview of the effectiveness of SC therapies in the management of TBI. To provide additional insights, we discuss the pathophysiology of TBI to elucidate the difficulty of transferring these types of therapies to clinical trials. An attempt is made to provide indicators of why the results obtained to date are not as promising as those obtained in preclinical studies. For this purpose, we collected and analyzed the clinical trials that use SC transplantation as a therapeutic treatment in patients with TBI. Finally, we offer our knowledge and experience in preclinical and clinical research to try to understand the current state of SC research to treat the sequelae of TBI.

## 2. Traumatic Brain Injury

It is well known that the pathophysiologic mechanisms of TBI are poorly understood, and that the anatomy of the brain is uniquely complex with multiple cell types (neurons, astrocytes, oligodendrocytes, and microglia) and multiple subtypes of these cells [20]. At the beginning of the last century, Ramón y Cajal, who laid the foundations of modern neurobiology, postulated in his book *Degeneration and Regeneration of the Nervous System* (1913) “… once development was ended, the founts of growth and regeneration of the axons and dendrites dried up irrevocably. In the adult centers, the nerve paths are something fixed, ended and immutable. Everything must die, nothing may be regenerated. It is for the science of the future to change, if possible, this harsh decree” [21]. This note is widely quoted in the literature to remind us that we are still a long way from understanding the true nature of the brain [20].

TBI is defined as an impairment of brain function caused by mechanical damage. It represents one of the main causes of death and disability in individuals aged between 1 and 45 years [22] and the greatest cause of death and disability globally among all trauma-related injuries [2]. The most common causes of TBI include falls, car accidents, assaults, and sports-related injuries [23]. The primary causes of TBI vary by age, socioeconomic factors, and geographic region [2]. In developed countries, there is a higher peak among subjects in adolescence, and a second peak is observed in the elderly [2]. Furthermore, the incidence rates by gender show that males are at least twice as frequently affected as females [24].

TBI occurs in two main phases. The primary injury relates directly to the traumatic event that causes a mechanical breakdown of brain tissue and results in direct neural cell loss, predominantly exhibiting necrotic death. The secondary lesion starts a few hours after the traumatic event and represents the main cause of the worsening in the evolution of TBI [12,25]. The secondary event comprises a complex cascade of cellular and molecular events, including excitotoxicity, oxidative stress, mitochondrial dysfunction, blood–brain barrier (BBB) disruption, and inflammation, which are responsible for further death/necrosis of brain tissue [12,26]. Interruption of normal cerebral blood flow (CBF) occurs within 24 h of trauma. The loss of self-regulation of blood flow increases intracerebral pressure (ICP) and decreases cerebral perfusion pressure (CPP) and CBF [27].

The immediate impact can cause two types of primary injuries: focal, which affect a specific area of the brain, or diffuse, which cause DAI. In addition, the impact may be a penetrating wound (open) or a closed-head injury (close). Open injuries involve a fracture of the cranial theca caused by a foreign object, whereas closed injuries are due to indirect impact without the entry of any foreign object into the brain. Most of the TBIs observed are of the closed type; however, a small percentage is represented by open TBI [22].

The severity of the initial injury is clinically identified by the Glasgow Coma Scale (GCS). The GCS is a neurological rating scale used to assess a patient’s level of consciousness after a head injury. This scale allows evaluation of visual, verbal, and motor functions. The scoring system is based on three types of response to stimuli: best eye-opening (maximum four points), appropriate and consistent verbal response (maximum five points), and best motor response (maximum six points). The sum of these scores yields a score between 3 and 15 [28]. According to GCS, TBI is classified as mild (13–15), moderate (9–12), or severe (3–8) [22,29].

The secondary degenerative processes are determined by the severity of the initial blow and the immediate pathophysiologic changes in the brain. After TBI, in the acute period, neurons and axons continue to die. The breakdown of the BBB and damage to the vascular endothelium allows blood components to leak into the brain parenchyma, including peripheral immune cells, which then contributes to the pro-inflammatory environment [9,20]. Astrocytes swell and tissue edema occurs. If edema is not controlled, ICP increases, which can lead to compression of arteries and decreased CBF. Cerebral ischemia commonly occurs under these conditions, leading to a vicious cycle of increasing edema, increasing ICP, and increased ischemia that can lead to death [20].

The initial injury induces an inflammatory response in order to fight infection and promote wound healing. Thus, head injury is also considered to be an inflammatory and immunological disease, rather than only a pure traumatological, neurological, or neurosurgical entity [30]. The inflammatory response includes complement activation, which accompanies the recruitment of inflammatory immune cells across the BBB [30,31]. These immune cells secrete prostaglandins, free radicals, and proinflammatory mediators, and increase expression of chemokines and cell adhesion molecules. This situation finally leads to an increase in the infiltration of immune cells into the damaged brain parenchyma [12,31] and augments the damaging consequences of the inflammatory response [32].

Following TBI, microglia proliferate and migrate to the site of the injury. Microglia work to remove the cellular debris at the lesion site, and produce cytokines and chemokines that activate pattern recognition receptors to bind damage-associated molecular patterns, and attract and polarize peripheral immune cells [20]. Therefore, the activated microglia manifest as different functional phenotypes, which are the subject of constant debate and study [33]. Microglia could provoke a protective response following TBI that limits the spread of damage and promotes recovery; however, they may also become pro-inflammatory by releasing neurotoxic molecules and proinflammatory cytokines, resulting in secondary damage [34]. Thus, microglia play a critical role in the neuroinflammatory response to TBI and could serve as a target for future research [35,36].

The immediate pathological consequences of these injuries cause ischemic events, similar to those observed with cerebral ischemia. They are responsible for changes in ion flux across the cell membrane, excitotoxicity, loss of ATP, lactate production, induction of cortical spreading depression, cytokine production, loss of barrier function at the BBB [20,37,38,39], and cerebral edema [40], and consequent alteration of oxidative phosphorylation, which leads to the accumulation of lactate [22].

The beginning of these processes triggers a series of mechanisms that lead to cell death directly or indirectly through the activation of the apoptotic process. They are also responsible for the production of reactive oxygen species (ROS) [41], and the oxidative stress plays an important role in the pathogenesis of TBI damage. The production of ROS has been shown to exacerbate the neurodegeneration process [42,43,44].

Unfortunately, if inflammation is not resolved it can become chronic [45]. In the aged brain, there is increased recruitment of peripheral macrophages into the TBI brain [46]. Furthermore, this chronic inflammation is associated with continued behavioral deficits [20]. This long-term inflammatory cascade initiated by TBI may persist and become amplified in the brain, predisposing TBI patients to neurodegenerative disorders and neurobehavioral deficits, and other mental health disorders [10,11,47,48].

The pathophysiological sequelae of TBI are highly complex and far from being sufficiently understood. Moreover, there is a still a significant lack of effective therapies to treat this terrible disease.

## 3. Stem Cell Therapy in Traumatic Brain Injury

Currently used interventions to improve the lives of people with TBI—including drug treatments, surgeries, and rehabilitation therapy—provide poor outcomes [17]. Our understanding of the use of SCs for TBI has accrued over in recent decades. Furthermore, many preclinical studies have been conducted about the use of cell therapy to treat TBI with encouraging results.

SCs are cells that show the multipotent capacity to differentiate toward different cell types and possess the capacity to renew themselves [49]. These cells would be able, firstly, to release neurotrophic factors to restore damaged neurons, and secondly, to regenerate damaged nerve tissue through differentiation or transdifferentiation into mature neural cells. Specifically, we refer to NSCs, iPSCs, and MSCs—such as BM-MSCs, AD-MSCs, and UC-MSCs—because they appear to be capable of regenerating damaged nerve tissue, as has been demonstrated in several in vivo studies using TBI animal models.

### 3.1. Neural Stem Cells

Multipotent neural progenitors and NSCs can be isolated from either embryonic or adult brain tissue, or be induced from both mouse and human embryonic SCs (ESCs). These cells proliferate in vitro through many passages without losing their multipotentiality [50].

The first experimental studies used fetal tissue or ESCs [51,52]. However, deriving clinical applications in patients from these studies is a challenge due to logistical, immunological, and ethical reasons. Sinson et al. evaluated the histological and behavioral impact of fetal neural transplantation with and without neurotrophin infusion in rats subjected to TBI [53]. This study demonstrated that fetal cortical cells transplanted in the injured cortex of rats could improve both posttraumatic cognitive and motor function, and interact with the injured host brain. The therapeutic effects of fetal cell therapy, and their contribution to the formation of additional neurons following transplantation, could additionally be enhanced through infusion of neural growth factor (NGF).

NSCs were first reported in their derivation from the embryonic mouse brain [54] and later were isolated from brain trauma patients [55,56]. NSCs have the potential for self-renewal and proliferation, and have been demonstrated to also differentiate into neurons, in addition to oligodendroglia and astrocytes [57].

Other studies involved pre-differentiating SCs increase survival, integration, and differentiation of transplanted cell into the adult central nervous system (CNS). These results indicate that lineage-restricted CNS precursors are well suited for transplantation into the adult CNS and provide a promising cellular replacement candidate [58,59]. In addition, the grafted precursors express the mature neuronal and glial markers, and the synaptic marker synaptophysin [59].

Preclinical works using NSCs in TBI rat models showed that human NSCs (hNSCs) are capable of surviving engraftment and differentiating into neurons which, in turn, correlate with improvements in neurological recovery [19,60]. Transplanted NSCs can survive in the traumatically injured brain, differentiate into neurons and/or glia, and attenuate motor dysfunction after TBI [61]. Mouse NSC transplants can survive upwards of 14 months, contributing to long-term improvements in memory, and may play a role in trophic support following TBI [62]. Additionally, other studies demonstrated that long-term cultured and cryopreserved hNSCs are suitable for transplantation and produced similar improvements in neurological recovery [63,64]; nonetheless, some aspects related to long-term cognitive and memory improvements are lacking [65]. Further, NSCs are thought to mediate their effects through cell replacement via differentiation into neurons in the injured region, and through secretion of glial cell-derived neurotrophic factor (GDNF) and other neuroprotective factors [66]. They are associated with the upregulation of synaptophysin and brain derived-neurotrophic factor (BDNF) expression, and can potentially repair and integrate neurons and glial cells at the injury site [67].

Another line of research studied the potential effectiveness of NSCs engineered to secrete neurotrophic factors such as NGF, BDNF, GDNF, and insulin growth-factors-1 (IGF-1). NSCs retrovirally transduced to produce NGF can markedly improve cognitive and neuromotor function and rescue hippocampal CA3 neurons when transplanted into the injured brain during the acute posttraumatic period [68]. Transplantation of GDNF-expressing neural progenitor cells (NPCs) in the acute posttraumatic period promotes graft survival, migration, and neuronal differentiation, and improves cognitive outcomes following TBI [69].

Thus, the use of immortalized cell lines represents an alternative strategy due to their enormous potential to improve functional outcomes after TBI in rodents, but raises serious safety concerns about the use of such cells in humans due to the risk of tumorigenesis [50,70,71].

### 3.2. Induced Pluripotent Stem Cells

In an effort to reduce the use of embryonic or fetal tissue due to ethical and viable sample collection issues, a number of studies have focused on induced pluripotent SCs (iPSCs) derived from adult somatic cells. Obtaining of iPSCs is usually undertaken according to the classical method described by the Yamanaka group from human fibroblasts [72,73]. Direct reprogramming of human fibroblasts into functional neurons in vitro could provide a source of patient-specific functional neurons for cell therapy [74], which would increase the cell sources for replacement of SCs.

Different preclinical studies have tested SCs possible application in animal models of TBI. Dunkerson et al. showed that combined therapy with enrichment environment and iPSCs therapy is more effective than only one of these approaches to improve cognition and motor performance [75] in rats after TBI. Other studies showed motor function improvement and the survival of human iPSCs in the host mouse brain tissue [76], and the migration of the implanted iPSCs to the injured brain areas from the injection site [77].

To date, the functional effect achieved for these cells in the treatment of experimental CNS injuries has been very limited. In addition, their clinical use raises many problems because of the difficulty of obtaining iPSCs, high therapy costs, and technique limitations.

### 3.3. Mesenchymal Stem Cells

As a source of SCs, MSCs appear to have received the greatest amount of attention in preclinical studies. Originally, MSCs were isolated from bone marrow where they support hematopoiesis [78]. MSCs are multipotent stromal cells with self-renewal capacity [79]. They can be easily harvested from a variety of tissue sources, such as bone marrow (BM), umbilical cord (UC), adipose tissue (AT), the placenta, and the oral cavity [78]. Their significant potential may explain why they appear to be efficacious for treating a wide range of different injuries and diseases [20].

The Mesenchymal and Tissue Stem Cell Committee of the International Society for Cellular Therapy (ISCT) proposes minimal criteria to define human MSCs (hMSCs) [80]. First, MSCs must be plastic-adherent when maintained in standard culture conditions. Second, MSCs be positive for CD73, CD90, and CD105, and negative for CD45, CD34, CD14, CD11b, CD79a, and CD19, and for the major histocompatibility complex class II (MHC-II) surface molecules [81]. Third, MSCs are potentially capable of differentiating into a variety of cell types including osteogenic, adipogenic, and chondrogenic [82]. In addition, it is known that MSCs have the ability to differentiate to neural lineages [82].

The therapeutic effects of MSCs are linked to their ability to modulate the inflammatory response and secrete neurotrophic factors that promote neurogenesis and angiogenesis, activating survival pathways and inhibiting apoptotic pathways, thereby enhancing neuroplasticity through neurite outgrowth and synaptogenesis [18,20].

Because of their pleiotropic characteristics, MSCs have been shown to improve neurological recovery in multiple CNS injury models, including TBI [83,84]. Recent studies regard MSCs as a potential candidate for cellular therapy of TBI and, thus, as an attractive alternative for embryonic and fetal SCs.

#### 3.3.1. Umbilical Cord-Derived Mesenchymal Stem Cells

Of growing interest are MSCs derived from human UC. The collection of UC-MSCs is noninvasive, and these cells are obtained easily and display strong self-renewal and differentiation abilities [85]. They have unique properties, such as high proliferation rates, wide multipotency, and hypoimmunogenicity; in addition, they do not induce teratomas and could have anticancer properties [86]. Further, they grow more quickly in vitro and may synthesize different cytokines [87]. UC-MSCs are a stromal population because they display the characteristics of MSCs [87]. It is easy to obtain a substantial number of UC-MSCs after several passages and extensive ex vivo expansion [88]. Further, UC-MSCs show a gene expression profile similar to that of embryonic SCs [85,86]. In summary, UC-MSCs are easy to harvest, store, and transport, and have a number of advantages, such as low immunogenicity power, less risk of rejection after transplantation, and lack of ethical controversy [89]. UC-MSCs are now proposed as a possible versatile tool for regenerative medicine and immunotherapy because they can accumulate in damaged tissue or inflamed regions, promote tissue repair, and modulate the immune response [90].

Few preclinical assays have been performed to test UC-MSCs in TBI. Zanier et al., 2011, showed that human UC-MSCs (hUC-MSCs) stimulate the injured brain and evoke trophic events, microglia/macrophage phenotypical switch, and glial scar inhibitory effects that remodel the brain and lead to significant improvement of neurologic outcomes. These cells confer trophic support by secretion of neutrophil activator, neurotrophin-3 (NT-3), BDNF, vascular endothelial growth factor (VEGF), and fibroblastic grow factor (FGF) to induce neuronal regeneration and vascularization in the damaged area [91]. In addition, when administered intravenously, they are able to reduce motor and neurological deficits in TBI rat models. The cells preferentially enter in the brain, migrate into the parenchyma of the injured brain, and express neuronal and glial markers [92].

Transdifferentiated and untransdifferentiated MSCs have shown therapeutic benefits in CNS injury. However, it is unclear which would be more appropriate for transplantation. To address this question, untransdifferentiated hUC-MSCs and transdifferentiated hUC-MSCs were transplanted into a rat model of TBI. The results showed that untransdifferentiated hUC-MSCs are more appropriate for transplantation and their therapeutic benefits may be associated with neuroprotection rather than cell replacement [93].

In the future, these cells could also be used within the field of genetic engineering. Yuan et al., 2014, studied the effect of BDNF gene-modified UC-MSC transplantation on neurological functional improvement in rats after brain trauma, and suggested that the cells can improve the neurological functions of rats with TBI [94], providing a therapy option with a multitude of applications.

In addition, UC-MSCs could have anti-inflammatory properties [90]. As a treatment for stroke, UC-MSCs have demonstrated the ability to reduce injury infarct volume and improve neurological recovery, which correlated with reductions in both systemic and neuroinflammation. These results suggest that restorative effects observed with these cells following ischemic brain injury may be mediated by trophic actions that result in the reorganization of host nerve fiber connections within the injured brain [95]. Further, they may act by inhibiting immune cell migration into the brain from the periphery and possibly by inhibition of immune cell activation within the brain [96]. Due to similarities between the events that occur after a cerebral infarction and TBI, the ability for UC-MSCs to modulate inflammation and improve recovery in cerebral injury models suggests this may be a promising avenue to explore for the treatment of TBI patients.

#### 3.3.2. Bone Marrow-Derived Mesenchymal Stem Cells

Another alternative to embryonic and fetal SCs are BM-MSCs. As a source of SCs and potential candidate for cellular therapy of TBI, BM-MSCs have been the subject of a greater number of bibliographic publications than other forms of SC. BM-MSCs are a heterogeneous population of cells, which provide support for hematopoietic cells. They have shown the ability to differentiate into bone, cartilage, and adipocytes [79]. It is also known that BM-MSCs show a low expression of MHC-II. Therefore, these cells have low antigenicity, which is one of the main advantages in the consideration of cell therapy protocols [97]. Among the different types of heterogeneous cell are a population of cells known as multipotent adult progenitor (MAP) cells, which proliferate indefinitely in vitro with a high capacity for proliferation and differentiation [98,99,100,101].

In recent years, much controversy has existed about the actual ability of BM-MSCs to differentiate into neuronal and glial cells, and the actual viability or usefulness of these cells. At the beginning of century, a series of studies suggested that BM-MSCs could undergo in vitro transdifferentiation when the medium is treated with different chemical agents, resulting in cells with adult neuronal-like morphology [102,103,104,105,106,107]. Several authors have questioned whether this is true neuronal transdifferentiation of the BM-MSCs, because the transformation could be temporal due to nonspecific changes of the cell cytoskeleton. The truth is that there is increasing evidence, both in vitro and in vivo, that it is possible to transform BM-MSCs into neurons and glial cells [108,109,110,111,112,113].

Based on their differentiation potential and accessibility, BM-MSCs have been studied in a large number of preclinical trials. The research group of Mahmood reported numerous experimental studies using BM-MSCs for the treatment of TBI. They provided further evidence about the possibility of reversing functional deficits in adult rats subjected to TBI through the administration of BM-MSCs when cells were administered intracerebral, intravenously, or intra-arterially [114,115,116,117,118,119,120,121,122,123,124]. There were able to demonstrate that some of the transplanted MSCs expressed neuronal and astrocytic markers in vivo, but few cells survived. However, these studies generally show a discrepancy between the positive results obtained and the low rate of cell transdifferentiation, suggesting that the effect of stromal cells, at least in part, may be due to the release of neurotrophic factors that induce regeneration in the host tissue [101].

Because the effects of BM-MSCs seemed independent of their ability to form new neurons in the damaged region of the brain, researchers have begun exploring the trophic effects of BM-MSCs. Neurotrophic factors promote neuronal survival and stimulate axonal growth. Thus, taking into account that trophic support provided by transplanted cells could play an important role in the treatment of damaged tissue, different studies showed the possibility of BM-MSCs to produce neurotrophic factors in culture. The results obtained show a high expression of growth factors, cytokines, and extracellular matrix molecules by the BM-MSCs [125,126]. The results were confirmed with human cells; TBI-conditioned human BM-MSC (hBM-MSC) cultures demonstrated a time-dependent increase in BDNF, NGF, VEGF, and hepatocyte growth factor (HGF), indicating a responsive production of these growth factors by the hBM-MSCs [127]. Therefore, the ability of secretion is determined by the surrounding microenvironment and self-growth status [126].

These results have been confirmed to several in vivo studies. The results confirm the ability of the cells to inhibit apoptosis, promote angiogenesis, and stimulate host progenitor cells to differentiate toward neurons and astrocytes. In this manner, BM-MSCs showed the ability to repair the lesioned tissue and recovered function in animal models of TBI [120,123,128]. Furthermore, BM-MSCs have been shown to penetrate the BBB, migrate to the site of injury, and, in addition to secreting various growth factors, regenerate the BBB [121,123].

Re-establishing blood flow to the injured area is critical for cellular growth and recovery. Following TBI, VEGF and other vascular growth factors are reduced. Multitudes of studies have demonstrated that BM-MSCs have an angiogenic effect in promoting neurologic recovery in TBI animal models. Hu et al. found that BM-MSC transplantation could increase the number of endothelial progenitor cells (EPCs) in the peripheral blood of rats with TBI, and the expression of peripheral angiogenetic markers and neuronal markers. The neurological function in transplanted group improved significantly compared to the controls [129]. Guo et al. demonstrated that BM-MSCs administered intravenously post-TBI upregulated VEGF and angiogenin-1 in a rat TBI model. These changes were correlated with the formation of micro-vessels [130]. Studies conducted in our lab using BM-MSCs showed that BM-MSCs could regenerate the injured spinal cord and produce newly formed nervous tissue. An important detail was to test how BM-MSCs also differentiate into cells that form new vessels and promote angiogenesis [109,111]. All of these results emphasize the importance of angiogenesis in improving neurologic outcomes after TBI and the role of BM-MSCs in facilitating this restorative process. We found a large number of reviews regarding the paracrine mechanisms of repair of the BM-MSCs [131,132]. Recent years have seen increased interest in MSC-derived exosomes as the paracrine source of neuroprotection, and their contribution to improve cognitive function and reduce inflammation [133,134].

In addition to promoting angiogenesis, BM-MSCs have been shown to ameliorate neuronal dysfunction induced by TBI through promotion of survival and normal growth of surviving neurons. Although neuronal cell death is a major contributor to poor neurologic outcome post-TBI, TBI not only induces cell death in immature granular neurons, it also causes significant dendritic and synaptic degeneration [60]. These observations point to a potential anatomic substrate to explain, in part, the development of post-traumatic memory deficits [135]. Addressing this cellular dysfunction in surviving neurons can help improve neurologic recovery. A study by Feng et al. found that administration of BM-MSCs via tail vein injection in a rat TBI model promoted neuromotor recovery via up-regulation of neurotrophic factors (VEGF and BDNF) and synaptic proteins (synaptophysin) in the brain. Thus, BM-MSCs not only helped restore the synaptic function of surviving neurons but also promoted neuro-regeneration [136]. Furthermore, therapy with hBM-MSCs during the acute phase of TBI suggested that increased levels of neurotrophic factors in the injured hemisphere lead to decreased neuronal apoptosis and enhanced neurological functional outcomes [137].

Regarding the long-term therapeutic efficacy of BM-MSCs, Mahmood et al. found that rat BM-MSCs injected intravenously one-week post-TBI in a rat model survived in the recipient animal at least three months post-treatment. In addition, functional improvements and growth factor production continued to be observed at three months post-BM-MSC administration [120]. Because improvements persisted despite declining numbers of BM-MSCs, this study highlights that functional recovery may not be directly correlated with BM-MSC graft survival.

Researchers have also investigated the migration capabilities of BM-MSCs to understand where the cells home in on injured tissue and whether BM-MSCs can be therapeutically applied with minimally invasive techniques. Research has shown that following intra-arterial infusion in rats with TBI, BM-MSCs migrated to the brain, but no functional recovery was observed, possibly because ligation of the internal carotid artery may have induced hypoperfusion, unintentionally exacerbating the initial injury [115]. Other studies used intravenous [114,116,119,120,121,122,123,124,129,130,136] or intracerebral [117,118] BM-MSC delivery in TBI models. Intravenous administration of BM-MSCs showed cell migration to the parenchyma of the injured brain and was associated with functional recovery and increase in the expression of growth factors, migration, proliferation, and angiogenesis, whereas intracerebral administration of BMSCs is associated mainly with glial and neural differentiation of transplanted cells.

To the best of our understanding, the biggest criticism of these experiences lies in the fact that, in almost all studies, BM-MSCs are administered at an early stage after TBI. In these studies, cell therapy was used in the early phases after trauma and a significant reduction in neurological deficits was observed [101]. Studies undertaken in our lab using BM-MSCs reported the effectiveness of cell transplants in chronic state, both in animal models of spinal cord injury (SCI) [108,109,111,112], and TBI [113,138,139,140,141]. The influence on neurological behavior after the cell therapy may be due to number of cells, route of transplantation, or transplantation technique.

#### 3.3.3. Adipose-Derived Mesenchymal Stem Cells

AT has emerged as an attractive cell source in tissue engineering and regenerative medicine because it can be easily collected and is enriched with stem/progenitor cell populations. AD-MSCs are isolated as part of the aqueous fraction derived from enzymatic digestion of lipoaspirate (the product of liposuction). This aqueous fraction, a combination of AD-MSCs, EPCs, endothelial cells (ECs), macrophages, smooth muscle cells, lymphocytes, pericytes, and pre-adipocytes, among others, is what is known as the stromal vascular fraction (SVF) [142].

AD-MSCs are obtainable in large quantities, under local anesthesia, with minimal discomfort. Human AT, which is obtained by suction-assisted lipectomy (i.e., liposuction), is processed to obtain a fibroblast-like population of cells. These cells can be maintained in vitro for extended periods with stable population doubling and low levels of senescence. AD-MSCs differentiate in vitro into adipogenic, chondrogenic, myogenic, and osteogenic cells in the presence of lineage-specific induction factors [143] and express several proteins consistent with the neuronal phenotype-like neurons [144,145].

AD-MSC transplantation has been shown to improve motor activity in an animal model of TBI, suggesting that these cells might be considered for patients with TBI. Topical application of AD-MSCs can improve functional recovery in an experimental TBI model. After treatment, neuronal death was reduced. The transplanted cells showed potential to modulate inflammation processes and differentiated into neural cells by co-expression of glial fibrillary acidic protein (GFAP), and Nestin and NeuN proteins [146,147]. The effects have not only been studied in an acute period; human AD-MSCs (hAD-MSCs) administered early and delayed after TBI showed significant improvements in neurocognitive outcomes and a change in neuroinflammation one month after injury [148].

Similar to other SC populations, it was initially thought that the main potential of AD-MSCs for regenerative medicine approaches was intimately related to their differentiation capability. These cells have the capacity to release important growth factors for wound healing, modulate the immune system, decrease inflammation, and home in on injured tissues [149]. AD-MSCs release neurotrophic factors with neuro-protective properties such as BDNF and GDNF [150]. This effect is related to the AD-MSCs’ secretome and the soluble factors found within it, which affect the protection, survival, and differentiation of a variety of endogenous cells/tissues [150]. Thus, AD-MSCs display cytokine secretory properties similar to those reported for BM-MSCs [151]. The secretome of AD-MSCs offers a series of immunoregulatory properties and is regarded as an effective method of mitigating secondary neuroinflammation induced by TBI. The secretome of AD-MSCs after TBI improves the neurological function and decreases TBI-induced neuroinflammatory environments that caused edema, apoptosis of neural cells, and nerve fiber damage [152].

Nevertheless, we could find studies in the literature about the lack of effects on TBI deficits after AD-MSC treatment. Kappy et al. described no differences in functional recovery after intravenous administration of AD-MSCs 3 h after TBI in rats, but showed increase in cell survival, decreased inflammatory marker release, and decreased evidence of neural injury in damaged tissue [153]. In addition, Dori et al. provided evidence about AD-MSC survival and migration into the periventricular striatum, but not about differentiation of AD-MSCs for neuronal or glial cell lineages. In this study, AD-MSCs stimulated proliferation of endogenous NSCs in the brain neurogenic niches, subventricular zone (SVZ), and hippocampal dentate gyrus (DG), and promoted a significant reduction in the lesion area, in addition to altering the post-injury pro-inflammatory profile of microglial and astrocytic cell populations [154].

SVF-derived mesenchymal progenitor/SCs can be easily expanded in vitro and have the potential to create diverse lineages of cells. Although there have been issues related to their isolation and purification, SVF cells demonstrate regenerative potential in damaged tissues or organs through paracrine and differentiation mechanisms. Furthermore, SVF cells augment immunological tolerance by promoting inhibitory macrophages and T regulatory cells, and by decreasing ongoing inflammation. Numerous implantations of freshly isolated autologous adipose tissue-derived SVF cells in cosmetic surgeries, and in a wide variety of other specialties, support the safety of SVF cells and have accelerated their clinical application. Despite these attractive advantages of SVF cells in clinical interventions, to our knowledge the recent status of clinical studies of various diseases has not been fully investigated [155].

The advantages of SVF over AD-MSCs are believed to lie in two fundamental areas. Firstly, although similar in properties such as immunomodulation, anti-inflammation, and angiogenesis, the distinctive, heterogeneous cellular composition of SVF may be responsible for the better therapeutic outcome observed in comparative animal studies. Secondly, unlike AD-MSCs, SVF is much more easily acquired, without the need for any cell separation or culturing conditions. Thus, the therapeutic cellular product is instantaneously obtained and has minimal contact with reagents, making it comparatively safe and subject to the fulfilment of fewer regulatory criteria. It should be noted that, whereas AD-MSCs find utility in both allogeneic and autologous treatments, SVF, due to the presence of various cell types known to cause immunological rejection, is suitable for autologous treatments only [142].

AD-MSCs are increasingly being investigated in clinical research. Nonetheless, numerous issues must be resolved before they can be established as a routine treatment in clinical protocols.

## 4. Role of Stem Cells in Traumatic Brain Injury: Clinical Studies

This review provides an overview of the studies recorded on http://clinicaltrial.gov (accessed on 15 October 2020) with autologous and no-autologous SC therapies, and the clinical trials approved by local ethics committees, relating to the treatment of TBI.

### 4.1. Autologous SC Therapies in Clinical Research

Table 1 shows the studies recorded on http://clinicaltrial.gov (accessed on 15 October 2020) with autologous SC therapies.

#### 4.1.1. NCT00254722

The first major cell therapy trials for TBI using BM-derived mononuclear cells (BM-MNCs) were conducted on children by Cox and colleagues in 2011 [156]. BM-MNCs are a heterogeneous mixture of immune cells and SCs including MSCs. The purpose of this study was to determine if BM-MNC autologous transplantation in children after isolated TBI is safe and will improve functional outcomes.

The completed phase 1 clinical trial NCT00254722 was performed on 10 patients (aged 5–14 years) with acute TBI (initial injury occurring less than 24 h prior to consent) diagnosed by GCS between 5 and 8. The research chose to use autologous BM-MNCs, among others reasons, because no in vitro culture/scaling methods for autologous application are necessary, the cells are readily available, there are no issues regarding uncontrolled replication (in contrast to embryonic or fetal cells), and no ethical objections exist regarding the cell type. Furthermore, they chose intravenous delivery because this approach avoided pulmonary the first-pass effect and due to the lack of a focal lesion, which makes stereotactic injection attractive. In addition, the catheter delivery systems were standard and the risks of selective cerebral catheterization and injection were avoided.

BM was harvested at 3 mL/kg of body weight performed between 12 and 30 h post-injury. The treatment was realized by single intravenous infusion of BM-MNCs at target dose by 6 × 10^6^ mononuclear cells/kg body weight, administered within 36 h of injury. Safety was determined by monitoring cerebral and systemic hemodynamics during BM harvest and BM-MNC transplantation. Values to determine infusion-related toxicity were measured through pediatric logistic organ dysfunction (PELOD) scores, hepatic enzymes, Murray lung injury scores (MLIS), and renal function. Conventional magnetic resonance imaging (MRI) data and neuropsychological and functional outcome measures were obtained at 1 and 6 months post-injury. There was no evidence of infusion-related toxicity of pulmonary, hepatic, renal, hematologic, or neurological organ systems. There were no deaths in the current study, but the authors determined that this was probably due to the applied exclusion criteria.

Further, with long-term (24 months) follow-up, they were able to estimate a treatment effect size on structural parameters (white and grey matter preservation) and functional/educational outcomes. MRI imaging comparing grey matter, white matter, and cerebrospinal fluid (CSF) volumes showed no reduction from 1 to 6 months post-injury. Dichotomized Glasgow outcome scale (GOS) at 6 months showed 70% with good outcomes and 30% with moderate to severe disability. However, although structural preservation and improved functional outcomes were observed, the study was underpowered and was not designed to provide conclusions regarding differences in treatment. Data from the study showed that there was no early or 6-month post-injury volumetric loss, and longer-term imaging will assess whether these findings are durable.

The first conclusion from this clinical research showed that BM harvesting and intravenous BM-MNC infusion, as a treatment for severe TBI in children, is logistically feasible and safe. Therefore, the authors proceeded with the implementation of controlled phase 2 trials, as discussed below (see NCT01851083).

#### 4.1.2. NCT01575470

Finalized clinical trials of BM-MNCs in adults with severe TBI have also been conducted [157]. Starting in 2012, the research group of Charles Cox undertook a cohort study of 1/2 Phase, in which 25 male and female adult patients (aged 18 years to 55 years) were enrolled with acute serious TBI (hospital admission GCS between 5 and 8 and initial injury occurring less than 24 h prior to consent).

This was a dose-escalation study in which 25 participants were assigned to five arms. A BM harvest was performed within 36 h of injury followed by a single intravenous infusion of autologous BM-MNCs. From each patient were collected 3 mL/kg of BM from anterior iliac crests. The first five subjects did not undergo the BM harvest procedure, although they were monitored and treated in the same manner as the other study participants, and completed all follow-up procedures. In the remaining three BM-MNC treatment groups, five patients received 6 × 10^6^ cells/kg, five patients received 9 × 10^6^ cells/kg, and five subjects received 12 × 10^6^ cells/kg in 0.9% saline containing 5% of human serum albumin. An additional control group was used to include participants across the time spectrum of the trials.

The goal of this trial was to assess the safety, feasibility, and efficacy of BM-MNC transplantation at the escalated doses. The primary hypothesis was that BM-MNC autologous transplantation after TBI is safe (harvest and infusion related toxicity), and the secondary hypothesis was that functional outcome measures will improve after BM-MNC infusion, reduce BBB permeability, offer neuroprotection, and preserve grey matter and white matter volumes.

Strict post-infusion monitoring therapy was performed during the first 7 h and whole blood analysis was realized every 12 h for the 7 days following cell infusion. Imaging protocol and neurobehavioral testing, in addition to functional outcome assessment, were performed at 1 and 6 months following injury. Additionally, inflammatory cytokines were measured in the plasma at baseline and 1 and 6 months after treatment. No events triggered the stopping rules and no serious adverse events related to the study protocol occurred.

There was no drop in the mean arterial pressure or CPP, or increase in ICP. None of the patients manifested severe side events after collection of BM or after BM-MNC transplantation. Treatment with BM-MNCs resulted in structural preservation of the corpus callosum and corticospinal tract, and these changes were correlated to neurocognitive outcomes; in addition, there was a reduction in the pro-inflammatory cytokine response to injury.

This study demonstrated that early BM-MNC harvesting and infusion is safe. There were no episodes of hypotension, hypoxia, or exacerbation of ICP/CPP parameters associated with harvest of BM or infusion of the cell product. There were no serious events in terms of organ failure. However, there did appear to be a dose-dependent pulmonary toxicity with an increase in the MLIS, suggesting a low-level lung injury. No patients developed hypoxia related to the infusion; however, because hypoxia adversely affects TBI outcomes, authors suggested it would be prudent to avoid any potential intervention that exacerbates pulmonary function.

The main conclusions of the clinical trial were: (a) early, autologous BM-MNC harvesting and infusion were safe and logistically feasible within a 36 h time window of treatment; (b) there was a treatment signal of brain tissue preservation measurable on a diffusion tensor MRI (DT-MRI) in a clinically relevant setting; (c) functional outcomes were correlated with brain tissue preservation; and (d) BM-MNC infusion could alter the global inflammatory response to injury as measured by cytokine profiles. A Phase 2b trial was planned to evaluate structural outcomes as the primary endpoint, eliminating the high-dose regimen (see NCT02525432).

#### 4.1.3. NCT01851083

In 2013, Charles Cox’s group began the follow-up trial NCT01851083 of the previously conducted Phase I trial NCT00254722 (see above) [156]. The investigators hope to determine the effect of intravenous infusion of autologous BM-MNCs on brain structure, and to evaluate the efficacy of cell therapy in neurocognitive and functional outcomes in children with severe TBI.

The study was designed as a Phase 2 safety/biological activity study and aimed to recruit 47 male and female patients. The inclusion criteria include, among others: between 5 and 17 years on the day of injury; GCS between 3 and 8 (best unmedicated post-resuscitation score during screening); and complete the BM-MNCs/Sham harvest and cell/placebo infusion within 48 h of the initial injury.

The patients were divided into three groups and received a single dose administered within 48 h from the time of injury. BM-MNCs were harvested and processed to obtain 6 × 10^6^ cells/kg or 10 × 10^6^ cells/kg body weight. Participants in the placebo group underwent a fictitious collection of BM and received a single intravenous infusion of 0.9% sodium chloride.

The primary outcome was to evaluate brain white matter and grey matter structural preservation on DT-MRI in the groups of patients treated and untreated after the trauma. DT-MRI quantitative indices of both macro- and microscopic integrity were evaluated and compared to DT-MRI of immediate post-injury treated and non-treated controls. The secondary outcome measures are white matter and grey matter preservation in regions of interest, and the improvement in functional and neurocognitive deficits 1 year post-infusion. Furthermore, authors assessed the infusional toxicity safety during 7 days after infusion. Laboratory and imaging studies were repeated at the 1-, 6-, and 12-month follow-up visits.

For this trial, no results are available yet.

#### 4.1.4. NCT02525432

The Phase 2b interventional study NCT02525432 started in 2015. The study continued the previous Phase I clinical trial NCT01575470 (see above), in which progenitor cell therapies showed promise in TBI by promoting CNS structural preservation, and reducing the neuroinflammatory response to injury [157]. The purpose of this study was to determine the effect of intravenous infusion of autologous BM-MNCs on brain structure and neurocognitive/functional outcomes after severe TBI in adults.

This is a dose-escalation placebo-controlled study designed to treat severe, acute TBI in adult patients and expected to recruit 55 male and female patients. The inclusion criteria are, among others, the following: adults 18 to 55 years of age on the day of injury with non-penetrating closed head trauma; GCS between 3 and 8 (best unmedicated post-resuscitation score during screening); and complete the BM-MNCs/Sham harvest and cell/placebo infusion within 48 h of the initial injury.

The patients will be divided into two groups. Thirty-three subjects will be treated with autologous intravenous transplantation of BM-MNCs within 48 post-TBI. The transplantation will start with the lowest dose of 6 × 10^6^ cells/kg body weight followed by a higher dose of 9 × 10^6^ cells/kg body weight within 48 post-TBI. In contrast, 22 control patients will undergo a fictitious collection of BM, in addition to treatment with a placebo infusion of saline.

The primary objective is to determine if the intravenous infusion of autologous BM-MNCs after severe TBI results in structural preservation of global grey matter volume and white matter volume and integrity, in addition to selected regions of interest in the corpus callosum. The subjects will be evaluated using MRI within 7–10 days at hospitalization, and 1 and 6 months after lesions. The secondary objectives are to determine if autologous BM-MNC infusion improves functional and neurocognitive deficits in adults after TBI and reduces the neuroinflammatory response to TBI; to evaluate spleen size and splenic blood flow over time using ultrasound and the corresponding changes in inflammatory cytokines; and to undertake infusion-related toxicity and long-term follow-up safety evaluations.

Subjects will be monitored closely for infusion-related toxicity and complications during the first 14 days post-infusion while also receiving the usual standard of care for TBI. Safety and outcome assessments will be performed at 1, 6, and 12 months post-injury study visits.

This study is still active.

#### 4.1.5. NCT02028104

This open label study of autologous BM-MNCs in TBI, NCT02028104, started in 2014 and finished in 2018 under the supervision of Alok Sharma. The purpose of this study was to study the effect of BM-MSC therapy on common symptoms in TBI. The study included patients with chronic TBI for greater than 6 months subject to intrathecal administration of autologous BM-MNCs. The results were published in 2020 [158], and a pilot study for this clinical research was published in 2015 [159].

The pilot study included 14 patients with chronic TBI that received neurorehabilitation and autologous BM-MNCs intrathecally. The inclusion criteria were diagnosed cases of chronic TBI and age above 1 year. Neurorehabilitation included physiotherapy, occupational therapy, speech therapy, and psychological intervention. The follow-up was performed at 1 week, 3 months, and 6 months after the intervention, and yearly thereafter.

The primary objective was to determine changes in clinical symptoms of TBI after cell therapy, and the secondary objective was to detect changes in the SF-8 Health Survey score and disability rating scale (DRS) after the treatment. The follow-up was undertaken at 6 months after the intervention.

These scales showed a positive shift in scores at the end of 6 months. Improvements were observed in various symptoms, in addition to the activities of daily living (ADLs). Authors observed significant symptomatic improvements, from one week to 6 months after intervention. Improvement was observed in fine motor skills, attention and concentration, and socialization skills. To a lesser extent, sensation and contractures/deformities and side facial muscle paralysis also showed improvement after intervention. The results suggest that cell therapy in combination with neurorehabilitation has the potential to reverse damage occurring in the brain after chronic TBI.

The results of this study suggest that cell therapy may promote functional recovery leading to an improved quality of life in chronic TBI. Although the results were positive, the improvements after cell therapy were not optimal. To elucidate this issue, the authors began the study NCT02028104 to establish cell therapy as a standard therapeutic approach.

The clinical study NCT02028104 was an extension from the previous pilot study [159], with a larger sample size of chronic TBI patients and a long-term follow-up to further establish the efficacy of intervention. An open label non-randomized clinical study was conducted to evaluate the safety and efficacy of intrathecal administration of autologous BM-MNCs in chronic TBI. The results were published in June 2020 [158].

The study was conducted in a single hospital center and included 50 patients diagnosed with chronic TBI with age above 1 year. The intervention included a combination of intrathecally transplanted autologous BM-MNC and neurorehabilitation. Neurorehabilitation included a personalized rehabilitation program based on individual needs, which comprised modalities, such as occupational therapy, physiotherapy, aquatic therapy, speech therapy, and psychological intervention. The longest follow-up of this study population was 71 months and no cell related adverse events were recorded, indicating long-term safety.

Overall, 92% of patients showed improvements in various symptoms, such as balance, voluntary control, memory, oromotor skills, lower limb activities, ambulation, trunk and upper limb activity, muscle tone, coordination, speech, posture, communication, psychological status, cognition, attention, concentration, and ADLs. In the study, 60% patients recorded an improved functional independence measure (FIM) score, suggesting improvement in ADLs such as bowel and bladder control, transfers, locomotion, communication, social cognition, and self-care activities. Reduced metabolism was seen in frontal, temporal, parietal, mesial temporal, occipital, basal ganglia, and cerebellar regions after the accident. These areas showed improved brain metabolism in the 10 patients who underwent a positron emission tomography (PET)–computed tomography (CT) brain scan 6 months after cell transplantation. These improvements correlated to the symptomatic changes observed in the patients at follow-up. Pediatric patients demonstrated better improvements in the objective scale than older patients (≥18 years). This outcome could be a result of the fact that brain plasticity is greater at younger ages and is malleable. The number of doses did not affect the outcome of transplantation.

One of the major limitations of the research was the lack of a control group to study the effect of cellular transplantation. However, improvements had plateaued in chronic TBI patients, all of whom were enrolled in rehabilitation prior to the intervention, and were not showing functional recovery. Following the combination of cellular transplantation and rehabilitation, the patients showed significant improvement, indicating that cell transplantation plays a vital role in the improvements.

In conclusion, this study demonstrated the safety and efficacy of cell transplantation in chronic TBI on long-term follow-up. Early intervention of cell transplantation at a young age in patients with mild TBI (mTBI) showed the best outcome in this study. When combined with neurorehabilitation, cell transplantation may help to improve the quality of life of patients with chronic TBI by recovering their lost functions and making them independent in ADLs. The clinical improvements seen in the patients correlated with the metabolic improvements on PET-CT scan post-cell transplantation. Hence, PET-CT scanning could be used to monitor the changes in the brain functions after cell transplantation.

#### 4.1.6. NCT02795052

The non-randomized clinical study NCT02795052 aimed to show if autologous BM-MSC transplantation by intravenous and intranasal administration provides an improvement in neurological functions in patients with some neurological conditions.

The study aims to recruit 300 male and female participants (aged over 18 years) with functional damage to the central or peripheral nervous system, including TBI, documented for least 6 months. The participants will be divided into two experimental groups. The first group will be treated via the BM-MSC intravenous route, whereas the participants in the second group will receive BM-MSCs via the intravenous and intranasal (lower 1/3 of nasal passages) route.

The primary outcome is to evaluate ADLs at 3, 6, and 12 months after the administration. The secondary outcome aims to assess deficits of neurologic function 3 to 12 months following the procedure, as identified by the patient as being impaired prior to treatment; as examples, neurologic functions may include speech, balance, hearing, gait, strength, pain, and paresthesia.

This study is still active.

#### 4.1.7. NCT02959294

The randomized Phase 1/2 study NCT02959294 aims to recruit 200 male and female participants (16–70 years of age) with TBI or concussion syndrome (CS). The purpose of this study is to examine safety and efficacy of parenteral introduction of adipose-derived SVF in patients with CS and TBI, and categorically examine the outcomes according to the elapsed time from the original concussive event. No delineation of those having recurrent damage and injuries will be made within this study.

The inclusion criteria include a documented history of mTBI, or TBI with correlated MRI or CT, with at less one month’s evolution. Patients must present symptoms associated with the injury, such as depression, cognitive disability, attention disorders, headaches or other persistent changes related to the lesion, and/or impaired social or occupational functioning following mTBI or TBI.

The study aims to identify improvement in long-term residua following adolescent and adult post-traumatic injuries often associated with contact sports and accidental causes; these are typically defined as reversible head injuries with temporary loss of brain function. The patients enrolled in the intervention arm will undergo a microcannula harvest of SVF.

The primary outcome relates to the measurement of participants with adverse events at baseline and 6 months after treatment, and cognitive changes in clinical symptoms (Montreal Cognitive Assessment Scale, MCAS) associated with concussion—TBI at 5 years after treatment. The secondary outcomes will be evaluated using Beck’s depression questionnaire (Beck’s Depression Inventory, BDI), and Adult Attention Deficit Assessment (Conner’s Adult Attention Deficit Disorder Rate Scale, CAADDR), both during the 5-year period after treatment. Finally, MRI brain studies will be conducted to evaluate progressive changes, comparing pre-treatment with 3- and 5-year status after treatment.

This clinical trial is still underway.

#### 4.1.8. NCT04063215

The final autologous clinical trial is NCT04063215. This study aims to determine the safety of Hope Biosciences AD-MSC (HB-adMSC) infusion and treatment effects on brain structure, neurocognitive/functional outcomes, and neuroinflammation after subacute and chronic neurological injury in adults.

The study aims to determine the safety and treatment effects of HB-adMSCs in adult patients with sub-acute or chronic neurological injury (including TBI). Inclusion criteria include adults between 18 and 55 years of age with documented head injury with functional neurological damage to the CNS unlikely to improve with present standard of care approaches; a GOS-Extended (GOS-E) score between 2 and 6; and onset or diagnosis of the injury or disease process greater than 6 months. In adult patients, 2 × 10^8^ HB-adMSCs will be infused three times over a six week period, spaced 14 days apart.

The primary outcomes will be evaluated by blood tests at baseline, and at 6 months and 1 year after infusion. The secondary outcomes involve an assessment of HB-adMSC infusion on global grey and/or white matter, and structural integrity of grey matter and white matter regions of interest in the corpus callous and corticospinal tracts, as measured in specific regions known to correlate with specific neurocognitive deficits in patients by MRI, at baseline and 6 months post-treatment. Moreover, several tests will be performed for both functional and neuropsychological assessments at baseline, after 6 months and 1 year post-transplantation. Finally, inflammatory cytokines will be assessed by cytometry analysis and monitoring the changes at 6 months and 1 year from baseline.

The final data collection is expected by August 2021.

### 4.2. Non-Autologous SCs Therapies in Clinical Research

An allogeneic SC-based therapy would provide an “off the shelf” treatment compared to autologous therapies, and, assuming similar safety and efficacy profiles, provide a more accessible treatment for patients. The only two clinical trials with allogenic SCs we found on clinicaltrial.gov (accessed on 15 October 2020) for the treatment of TBI are described below (Table 2).

#### 4.2.1. NCT02416492

In NCT02416492, the safety and efficacy of San Bio’s proprietary adult BM-MSCs genetically modified to express the intracellular domain of human Notch-1 (SB623 cells) to treat chronic TBI were studied. SB623 cells are adult BM-derived cells that are transiently transfected with a plasmid construct encoding the intracellular domain of human Notch-1.

A clinical trial of these cells in stroke patients demonstrated that cells were safe and induced significant motor function improvement in adults (NCT01287936) [160]. The study showed no serious adverse events likely attributed to SB623, and only minor adverse events, mostly grade 1 or 2 (with one grade 3), which were unrelated, unlikely to be related, or possibly related to SB623. No dose-limiting toxicities were observed.

The clinical study NCT02416492, started in 2015, is the first study with modified SC therapy to treat chronic TBI. The aim of the study was to evaluate the effectiveness, safety, and tolerability of the stereotactic intracranial implantation of allogeneic SB623 cells.

This is a controlled Phase 2 study, which enrolled 61 male and female subjects aged 18 to 75 years, with stable, chronic motor deficits secondary to focal TBI. Inclusion criteria include a documented history of TBI, with correlated MRI or CT of at least 12 months post-TBI, neurological motor deficit, and GOS-E score of 3–6 (i.e., moderate or severe disability). The participants were randomly divided into four groups. One of these did not receive the surgical intervention (control group) and three groups comprised patients who received 2.5 × 10^6^, 5 × 10^6^, or 10 × 10^6^ SB623 cells. The SB623 cells were surgically implanted adjacent to the injured cerebral region.

The primary purpose was to evaluate the clinical efficacy of intracranial administration of SB623 cells on patients with chronic motor deficit from TBI using the Fugl–Meyer Motor Scale (FMMS) from baseline to 6 months. Secondary purposes of the study were to: (1) evaluate the effect of intracranial administration of SB623 cells on disability parameters; and (2) evaluate the safety and tolerability of intracranial administration of SB623 cells. Changes in the DRS, Action Research Arm Test (ARAT), Gait Velocity, change from baseline in T scores of Neuro-QOL Domains, and global rating of perceived change scores were analyzed, at 6 months after treatment.

Results information has been submitted to clinicaltrials.gov by the sponsor or investigator, but is not yet publicly available (or “posted”) on the site.

#### 4.2.2. NCT02742857

The Phase 1 study NCT02742857 aims to recruit 20 male and female patients (15 to 65 years). This is a multi-modality study (using intrathecal bioactive peptides, SCs, laser and transcranial IV laser, and median nerve stimulation as adjuvants). The aim of the study is to show the possibility of reversal of brain death induced by TBI with DAI. Inclusion criteria include individuals declared brain dead from a TBI having DAI on MRI and not permitting organ donation.

The study was designed using different therapeutic interventions, two biological (MSCs transplantation and infusion of intrathecal bioactive peptides, BQ-A Peptide Extract), and two via a device (transcranial laser therapy and median nerve stimulation).

The primary outcome is to document reversal of brain death as noted in clinical examination or electroencephalogram (EEG) during a 15-day time frame. Secondary outcomes are to provide a CSF analysis of color consistency and cell counts, microbial evaluation, and MRI analysis to analyze any changes in meninges in the 15-day time frame. In addition, blood pressure, pulse rate, oxygen saturation, and respiratory changes in the 15-day time frame will be assessed.

To date, no information is available.

### 4.3. Clinical Trials Approved by Local Ethics Committees

In this section, clinical trials published in indexed journals are described (Table 3). Many of these studies report the results obtained from treatment of TBI with SC therapy and provide important efficacy and safety data.

A Phase I-II trial of autologous multipotent NPCs/NSCs implantation for the treatment of brain trauma was published in 2005 [55]. Authors implanted NPCs/NSCs into traumatic regions for eight patients with open brain trauma and used another eight untreated counterparts as case control. Within 2-year follow-ups, patients were investigated by functional MRI (fMRI), F-18-fluorodeoxyglucose PET (FDG-PET), somatosensory evoked potential (SEP), and DRS for functional recovery. In contrast to the control group, implantation of NSCs was associated with a significant improvement in the patients’ neurological function. The improved brain function was accompanied by partial recovery of activity in damaged areas as assessed by fMRI and SEP, and by significant increases in neural viability within injured territories as assessed by FDG-PET. Importantly, none of patients had seizures, fever, or deterioration of neurological function after cell implantation. In addition, injection of progenitor cells did not induce an acute inflammatory response as measured by blood examinations. Thus, the culture and expansion of NSCs followed by re-implantation appears to be feasible and safe in patients with open brain trauma.

Moreover, in 2005, the results of applying cell transplantation therapy in severely head-injured patients were presented [161]. The patients initially were in a state of coma (GCS score 3–7) due to TBI. Cells prepared from fetal nervous and hematopoietic tissues were grafted subarachnoidally via lumbar puncture. The control group consisted of 38 patients and was clinically comparable with the trial group. From the results obtained, it appears that cell transplantation treatment promoted both wakening consciousness of the patients and their following neurological rehabilitation. The death rate was also less after cell treatment. Importantly, no serious complications that might limit application of the cell-based technologies in head-injured patients were noted. The authors presented the results as being clearly promising and noted the possibility of applying cell therapy in severely head-injured patients as early as within the acute period of the disease, when the patient is unconscious.

In 2006, Zhu et al. reported the feasibility of labeling NSCs from humans with superparamagnetic iron oxide nanoparticles and tracking them with the use of MRI [56]. A 34-year-old man had brain trauma in the left temporal lobe. During an emergency operation, exposed neural tissue from his brain was collected and cultured to select NSCs. The cultured NSCs were incubated with a contrasting agent containing superparamagnetic iron oxide nanoparticles. These autologous cultured NSCs were then implanted stereotactically around the region of damage brain. One week after implantation, the change in signal was consistent with cell accumulation and proliferation around the lesion. The signal at the periphery of the lesion intensified during the second and third weeks, suggesting that the NSCs had migrated from the primary sites of injection to the border of the damaged tissue.

In 2006, the Seledtsov et al. [162] presented the results of a retrospective clinical study of the efficiency of cell therapy in the treatment of II–III degree comatose patients with severe brain injury. The main group consisted of 25 patients (8 women and 17 men) aged 18–63 years and a control group consisting of 25 patients aged 19–60 years (the severity of brain injury and prognosis of the disease course were comparable to those in the main group). Cell suspension consisting of cells derived from immature nervous and hematopoietic tissues was injected into the recipient subarachnoidal space through a cerebrospinal puncture. The mortality in the study group was 8% vs. 56% in the control group. The 1.5-year follow-up demonstrated significantly better quality of life in patients receiving cell therapy in comparison with patients of the control group. The study demonstrated the efficiency of cell therapy in patients with severe brain injury during the acute period of the disease but was ineffective for patients in a comatose state caused by hypoxic encephalopathy.

Zhang et al., (2008) introduced a combined procedure to deliver autologous BM-MSCs to patients with TBI [163]. The feasibility and safety of this procedure were assessed for seven TBI patients. Neurologic function was also evaluated. Six male patients, aged between 30 and 55 years, and one female patient, aged 6 years, were enrolled in this program. The inclusion criteria were as follows: TBI with or without paralysis; a Barthel index (BI) value less than 40; and a further operation required for the purpose of cranial correction or replacement. MSCs were isolated by BM aspiration and expanded in culture. A primary administration of 10^7^–10^9^ cells was applied directly to the injured area during the cranial operation. After an interval of 4–12 days, a second dose of 10^8^–10^10^ cells was infused intravenously. All patients were followed-up regularly for 6 months. There was no immediate or delayed toxicity related to the cell administration within the 6-month follow-up period. Neurologic function was significantly improved at 6 months after cell therapy. The procedure proved feasible and safe during a 6-month follow-up period. The MSCs’ homing efficacy was presumably enhanced through this combined cell delivery procedure.

A study was published in 2013 [164] to explore the clinical therapeutic effects and safety of autologous BM-MSCs therapy for TBI by lumbar puncture. The SCs were isolated from the BM of the patients and transplanted into the subarachnoid space by lumbar puncture. Fourteen days after cell therapy, no serious complications or adverse events were reported. Improvement in the function of brain after transplant, post-therapeutic improvements in consciousness, and improvements in motor functions were detected after therapy. The age of patients and the time elapsed between injury and therapy had effects on the outcomes of the cellular therapy. Young patients improved more easily than older ones. The main conclusion was that the earlier the cellular therapy begins in the subacute stage of TBI, the better the results.

Wang et al. conducted a study [165] to investigate the effects of transplantation of UC-MSCs in patients with sequelae of TBI, which had been sustained more than one year previously. Patients were diagnosed as having sequelae of TBI based on clinical manifestations, head CTs, and MRI examinations, and suffering from CNS dysfunction at the time of recruitment. Forty patients were randomly assigned to the SC treatment group or the control group. The patients in the SC treatment group underwent four transplantations via lumbar puncture. Patients were evaluated using FMMS and FIM before and at 6 months after the cell transplantation. The patients in the control group did not receive any medical treatment (i.e., neither surgery nor medical intervention), and their FMA and FIM scores were determined on the day of the visit to the clinic and at 6 months after that clinical observation. The study results confirmed that the UC-MSC transplantation improved the neurological function and self-care in patients with TBI sequelae. Furthermore, the efficacy and safety of the treatment with UC-MSCs in patients with TBI sequelae were shown.

Liao et al. [166] carried out a retrospective cohort study using data of patients that were enrolled in the Phase I trial NCT00254722 (see above) [156]. The aim of the study was to understand if BM-MNC transplantation within 48 h after trauma induced a reduction in treatment intensity for managing elevated ICPs relative to pediatric patients with severe TBI. Study patients (29) were aged 5–14 years with post resuscitation GCS scores of 5–8 and with trauma occurring <24 h. Ten patients were assigned to a treatment group and 19 to the control group. The treatment group received 6 × 10^6^ autologous BM-MNCs/kg body weight intravenously within 48 h of injury. The primary measure was the Pediatric Intensity Level of Therapy (PILOT) scale, used to quantify the treatment of elevated ICP. Secondary measures included the PELOD score and days of ICP monitoring as a surrogate for length of neurointensive care. In the treatment group, after transplantation, a significant reduction was observed in the PILOT score beginning at 24 h post-therapy compared to the control group. In conclusion, intravenous autologous BM-MNC therapy was associated with lower treatment intensity required to manage ICP, lower severity of organ injury, and lower duration of neurointensive care following severe TBI.

A Phase I trial of intravenous or intrathecal administration of autologous MSC-derived NSC-like cells was designed to test the safety and feasibility of this potential treatment in patients with severe TBI [167]. The study reported the observations in 10 patients that received intravenous or intrathecal injections of human NSC-like cells, and were evaluated with physical and neurological examinations, routine laboratory tests, and neuro-radiological findings. The results indicated that the majority of patients experienced improved neurological function to different degrees during the follow-up period. The procedure was tolerated relatively well and there were no significant adverse events due to the presence of the NSC-like cells in the vessel or subarachnoid space. Higher serum levels of NGF and BDNF were detected following the transplantation, compared with the levels prior to treatment. Furthermore, there was no evidence of tumor formation, venous thromboembolism, intracranial infection, or systemic infection in any of the patients following cell transplantation. Authors suggested that transplantation of autologous NSC-like cells is feasible and appears to be safe for the treatment of non-acute severe TBI.

We have used a cell therapy medicament (NC1), initially approved as a medicament under clinical investigation and recently approved for hospital use (registration number 83796), in different clinical trials. This treatment consists of autologous BM-MSCs and autologous plasma as its excipient. NC1 has been used for the treatment of sequelae of SCI and post-traumatic syringomyelia in clinical trials (clinicaltrials.gov identifiers: NCT01909154, NCT02165904, NCT02570932, and NCT02807142) [168,169,170,171,172]. Based on our experience, we solicited authorization for intrathecal administration of NC1 in three patients diagnosed with DAI [173]. FDG-PET-CT scans were performed. The total number of autologous BM-MSCs administered intrathecally were 60 × 10^6^ (one patient), 100 × 10^6^ (one patient), and 300 × 10^6^ (one patient). The three patients showed variable but significant clinical improvement after cell therapy. This improvement was associated with an early and progressive increase in brain glucose metabolism, measured using FDG-PET. Although preliminary, our present findings suggest a benefit of intrathecal MSCs in patients with established sequelae after TBI, and show that a strong and progressive increase in brain glucose metabolism can be obtained after this type of cell therapy. Given this, it is obvious that the development of clinical trials is necessary to confirm the potential benefit of intrathecal BM-MSC administration in DAI; thus, we began a clinical trial in 2018 (see below).

Finally, at the beginning of 2020, a clinical study was published relating to the ability of MSCs in oxidative stress neutralization. It is hypothesized that autologous transplantation of BM-MSCs decreases the range of oxidative stress in patients in minimally conscious state (mCS) [174]. The aim of this study was to evaluate the effect of BM-MSC transplantation on selected markers of oxidative stress in mCS patients. Antioxidant capacity was measured in CSF and plasma collected from nine patients aged between 19 and 45 years, remaining in mCS for 3 to 14 months. Total antioxidant capacity, ascorbic acid and ascorbate concentrations, superoxide dismutase, catalase, and peroxidase activity were analyzed, and the presence of tested antioxidants in the CSF and plasma was confirmed. For the purposes of the experiment, a diagnostic protocol based on MRI, fMRI, and EEG, and analysis of the 99mTc-hexamethylpropylene amine oxime (HMPAO) single photon emission tomography/computed tomography (SPECT-CT) cerebral perfusion factor, to assess the differentiation of consciousness disorders, were used. The results suggested that BM-MSCs modulate oxidative stress intensity in mCS patients, mainly via ascorbate and catalase activity.

**Table 3:** Clinical trials approved by local ethics committees.

References	Aim	Enrolment	Ages	Condition	Treatment	Conclusions
[55]	A Phase I-II trial to determine if autologous multipotent neural progenitor cells (NPCs)/NSCs implantation in patients with open brain trauma is feasible and safe	8 treated 8 controls	-	Patients with open brain trauma	Transplantation of NPCs/NSCs into traumatic regions	The culture and expansion of NSCs followed by re-implantation appears to be feasible and safe in patients with open brain trauma
[161]	Determine if cell therapy in severely head-injured patients as early as within acute period of a disease is safe	38 treated 38 controls	18 to 63 years	Severely head-injured patients	Cells preparation from fetal nervous and hematopoietic tissues transplanted subarachnoidally via lumbar puncture	The authors presented the results as promising and noted a possible rationality of applying cell therapy in severely head-injured patients as early as within the acute period of a disease, when patient is unconscious
[56]	Determine the feasibility of labeling NSCs from humans with superparamagnetic iron oxide nanoparticles and tracking them with the use of MRI in a patient with brain trauma	1	34	Brain trauma in the left temporal lobe	NSCs incubated with a contrast agent containing superparamagnetic iron oxide nanoparticles and stereotactically implanted around the region of damage brain	Stem-cell engraftment and migration after implantation could be detected noninvasively with the use of MRI
[162]	Results of a retrospective clinical study of the efficiency of cell therapy in the treatment of II-III degree comatose patients with severe brain injury	25	18 to 63 years	Severe brain injury in coma of II-III degree (GCS 3–5)	Cells derived from immature nervous and hemopoietic tissues injected into the recipient subarachnoidal space through a cerebrospinal puncture	The study demonstrated the efficiency of cell therapy in patients with severe brain injury during the acute period of the disease
[163]	Determine the safety and feasibility of a combined procedure to deliver autologous MSC to patients with traumatic brain injury	7	6 to 55 years	TBI with or without paralysis	MSCs isolated to bone marrow administrated intracranially and after an interval of 4–12 days infused intravenously	The procedure was feasible and safe during a 6-month follow-up period and the MSCs’ homing efficacy was enhanced through this combined cell delivery procedure
[164]	Explore the clinical therapeutic effects and safety of autologous BM-MSC therapy for TBI by lumbar puncture	97 treated 69 controls	-	Patients with a vegetative state or with disturbance in their motor activity after severe TBI for at least 1 month	Explore the clinical therapeutic effects and safety of autologous BM-MSCs therapy for TBI by lumbar puncture	No serious complications or adverse events, improvement in the function of brain after transplant, post-therapeutic improvements in consciousness, and improvements in motor functions were detected after therapy. The age of patients and the time elapsed between injury and therapy had effects on the outcomes of the cellular therapy
[165]	Investigate the effects of transplantation with UC-MSCs in patients with sequelae of TBI, which had been sustained more than one year previously	20 treated 20 controls	7 to 57 years	Patients with sequelae of TBI, which had been sustained more than one year previously	The patients in the UC-MSC treatment group underwent four transplantations via lumbar puncture	UC-MSCs transplantation improved the neurological function and self-care in patients with TBI sequelae and the efficacy and safety of the treatment with UC-MSCs in patients with TBI sequelae were shown
[166]	Understand if BM-MNCs transplantation within 48 h after trauma-induced a reduction in treatment intensity against managing elevated ICPs relative in pediatric patients with severe TBI	10 treated 19 controls	5 to 14 years	Patients with acute TBI (initial injury occurring less than 24 h prior to consent)	The treatment group received 6 × 10^6^ autologous BM-MNCs/kg body weight intravenously within 48 h of injury	Intravenous autologous BM-MNCs therapy was associated with lower treatment intensity required to manage ICP, lower severity of organ injury, and lower duration of neurointensive care following severe TBI
[167]	Test the safety and feasibility of intravenous or intrathecal administration of autologous MSCs-derived NSCs-like cell in patients with severe TBI	10 treated	18 to 65 years	Severe TBI within the past 60 days	Intravenous or intrathecal administration of autologous MSC-derived NSC-like cells	The majority of patients experienced improved neurological function. Higher serum levels of NGF and BDNF and no evidence of tumor formation, venous thromboembolism, intracranial infection, or systemic infection after cell transplantation were detected. Autologous NSC-like cell transplantation is feasible and appears to be safe for the treatment of non-acute severe TBI
[173]	Test the safety and feasibility intrathecal administration of autologous BM-MSCs in patients with severe TBI diagnosed with DAI	3 treated	30, 57, and 35 years	Patients with severe TBI diagnosed with DAI	Total number of BM-MSCs administered was 60 × 10^6^ (one patient), 100 × 10^6^ (one patient) and 300 × 10^6^ (one patient)	Benefit of intrathecal BM-MSCs in patients with established sequelae after TBI, and a strong and progressive increase in brain glucose metabolism after cell therapy
[174]	Evaluate the effect of BM-MSCs transplantation on selected markers of oxidative stress in mCS patients	9	19 to 45 years	Patients remaining in mCS for 3 to 14 months, as a result of a TBI, among others	20 × 10^6^ BM-MSCs injected intrathecally three times every two months	BM-MSCs modulate oxidative stress intensity in mCS patients, mainly via ascorbates and catalase activity

## 5. Status of Our Research about Cell Therapy in TBI

Although these findings appear promising, further studies are required. The size of the brain lesions and their variability appear to be determinants of the need to find alternatives to enhance cell survival and differentiation of the transplanted SCs. Similarly, the critical number of BM-MSCs that are necessary to restore the functional deficits after brain trauma represents one of the main issues to be resolved. We developed experimental studies trying to confirm the therapeutic efficacy of the administration of BM-MSCs during the chronic phase of severe TBI in which there is an established neurological deficit.

To investigate the utility of delayed transplantation of BM-MSCs to improve the neurological sequelae after TBI, we performed a number of studies to determine the best means of realizing the therapy—intracerebral [113,138,141], intravenous [139], or intrathecal [140] administration. After intracerebral administration, we showed that BM-MSCs survived in the host tissue, and some of these showed neuronal and astroglial markers. Furthermore, a significant increase in endogenous neurogenesis and improvement in neurological deficits was found [113]. However, in our experience from a rat model of TBI, intravenous [139] and intrathecal [140] BM-MSC transplantation failed for different reason. During the acute period after trauma, migration of transplanted BM-MSCs could be mediated by inflammatory cytokines and chemokines generated by the cerebral injury. In our model, cell therapy was performed after a long delay following trauma, by which time the factors released during the acute period were absent. However, similar to direct parenchymal injections, subarachnoid transplanted BM-MSCs survive, migrate to the injury cavity, and differentiate into mature neural cells at least 6 months after engraftment [140]. Once it is demonstrated that the cells colonize and integrate into the damaged tissue, optimizing the number of administered cells could lead to significant neurological recovery and trophic factor production. These results suggest the possibility that BM-MSCs administration through subarachnoid administration may be a treatment for TBI. We gathered evidence regarding the progressive functional recovery after transplantation of BM-MSCs three months after SCI in pigs [111], as confirmed in clinical trials (NCT01909154, NCT02165904, NCT02570932, and NCT02807142). These findings could be translated to chronic TBI and support the possible utility of subarachnoid BM-MSC transplantation in clinical trials. Furthermore, we suggested for the first time a relationship between scores of brain damage and effectiveness of cell therapy with BM-MSCs for the treatment of chronic TBI [138]. In our rat TBI model, BM-MSC therapy was more effective in moderate-TBI than in the severe-TBI transplanted group. These findings suggested that the severity of neurologic damage might determine the potential effect of cell therapy when applied to chronically established TBI.

Finally, brain lesion size and variability appear to be determinants in cell therapy efficacy. New viable strategies could be the use of biological matrices that allow cell survival and differentiation of transplanted SCs in greater proportions than in conventional therapy [101]. In 2018, we reported the study of platelet-rich plasma scaffolds (PRPS) as support for BM-MSCs in a delayed phase after severe TBI in rats [141].

In addition, based on our preclinical experience of chronic cell therapy of TBI and our clinical study of the intrathecal administration of the medicament NC1 in three patients diagnosed with DAI [173], we are currently performing a clinical trial of intrathecal administration of autologous adult BM-MSCs in DAI. This is a type 2 clinical trial (EudraCT number: 2017-001824-23) using the medicament NC1 (see above; autologous BM-MSCs and autologous plasma as its excipient). The main objective of the trial is to analyze the potential clinical efficacy of intrathecal administration of the medicament NC1, in the subarachnoid space, in the treatment of a homogeneous group of patients with established chronic cerebral injury and previous diagnosis of DAI. The secondary objective is to confirm the treatment safety of the dose raised from the present study. The inclusion criteria include a TBI background with cognitive effect and clinical diagnosis of DAI, age between 18 and 70 years, and the possibility of follow-up evolution and neuro-rehabilitation support during the follow-up period. The main assessment endpoints will be those related to the possible efficacy of the treatment, which will be measured by means of changes in GCS, DRS, Galveston Orientation, and Amnesia Test (GOAT), Ashworth and FIM scales, and their subsections, in addition to neurophysiological studies and PET-CT. Evaluation with the neurologic scales was realized at months 1 (baseline), 3, 6, 9, and 12 (final assessment), and the neurophysiological studies and PET-CT were realized at month 1 (baseline) and month 12 (final assessment).

The clinical trial is still underway and is expected to end this year.

## 6. Conclusions

TBI is a highly complex disease. At present, there are no known effective treatments able to reduce the consequences of the brain injury. In recent decades, cell therapies have been demonstrated as useful tools that could reduce the effects of TBI. These therapies appear to be safe and have demonstrated the ability to improve neurological and motor functions in TBI patients. However, the mechanisms by which these improvements are mediated remain unknown and the number of studies undertaken is small. It is necessary to identify the best route for the administration of cell therapy to achieve better survival of the transplanted cells after administration, and the optimal time for the application of cell therapy after trauma. Although unresolved issues remain, it is obvious that in recent years new techniques of cell therapy involving adult SCs, in conjunction with new concepts related to the possibility of regeneration of the adult nervous system have provided prospective new opportunities for the treatment of TBI. Any advance in this field, as in many other areas of neurobiology, will require close collaboration between basic and clinical researchers. Future studies are needed to elucidate the mechanisms by which SC therapies promote recovery following TBI, and to evaluate the effectiveness of these therapies. The awaited outcomes and future studies will be necessary to exploit the use of cell transplantation for the management of TBI.

## Figures and Tables

**Table 1:** Clinical trials of autologous SC therapies recorded on clinicaltrial.gov.

NCT Number/ References	Official Title	Aim	Phase	Enrolment	Ages	Condition	Treatment	Conclusions
NCT00254722 [156]	Safety of Autologous Stem Cell Treatment for Traumatic Brain Injury in Children	Determine if BMPC autologous transplantation in children after isolated TBI is safe and will improve functional outcome	1	10	5 to 14 years	Patients with acute TBI (initial injury occurring less than 24 h prior to consent)	Single intravenous infusion of 6 × 10^6^ mononuclear cells/kg body weight	Bone marrow harvest and intravenous mononuclear cell infusion as treatment for severe TBI in children is logistically feasible and safe
NCT01575470 [157]	Treatment of Severe Adult Traumatic Brain Injury Using Bone Marrow Mononuclear Cells	Determine if bone marrow harvest, BM-MNC separation, and re-infusion in adults with acute severe TBI is safe and will improve functional outcome	1/2	25	18 to 55 years	Patients with acute TBI (36 h time window of treatment)	10 controls 15 treatment groups at doses of 6 × 10^6^ cells/kg (low dose group), 9 × 10^6^ cells/kg (medium-dose group), and 12 × 10^6^ cells/kg (high dose group) in 0.9% saline containing 5% of human serum albumin	BM-MNC infusion in adults with severe TBI is safe and show potentially a CNS structural preservation treatment effect
NCT01851083 (continuation NCT00254722)	A Phase 2 Multicenter Trial of Pediatric Autologous Bone Marrow Mononuclear Cells (BMMNCs) for Severe Traumatic Brain Injury (TBI)	Determine the effect of intravenous infusion of autologous BM-MNCs on brain structure and neurocognitive/functional outcomes after severe TBI in children	2	47	5 to 17 years	Patients with severe TBI (infusion within 48 h of the initial injury)	Patients are divided into three groups, one group as control and two groups receiving 6 × 10^6^ cells/kg or 10 × 10^6^ cells/kg weight	Clinical trial active, no results are available yet
NCT02525432 (continuation NCT01575470)	Treatment of Adult Severe Traumatic Brain Injury Using Autologous Bone Marrow Mononuclear Cells	Determine the effect of intravenous infusion of autologous BM-MNCs on brain structure and neurocognitive/functional outcomes after severe TBI injury in adults	2	55	18 to 55 years	Patients with acute TBI (infusion within 48 h of the initial injury)	Patients are divided into two groups: 33 subjects treated with 6 × 10^6^ cells/kg body weight followed by a higher dose of 9 × 10^6^ cells/kg of body weight and 22 control patients	Clinical trial active, no results are available yet
NCT02028104 [158,159]	Open Label Study of Autologous Bone Marrow Mononuclear Cells in Traumatic Brain Injury	Study the effect of stem cell therapy on common symptoms in patients with TBI	1	50	1 to 65 years	Diagnosed cases of chronic TBI	Combination of autologous BM-MNCs intrathecally transplanted and neurorehabilitation	This study demonstrated the safety and efficacy of cell transplantation in chronic TBI on long term follow-up
NCT02795052	Neurologic Bone Marrow Derived Stem Cell Treatment Study	Determine if the autologous BM-MSC transplantation by intravenous and intranasal administration will provide an improvement in neurological functions in patients with some neurological conditions	-	300	Over 18 years	Participants with functional damage to the central or peripheral nervous system documented by least 6 months, including TBI	The participants are divided into two experimental groups. The first group is treated with the BM-MSC intravenous route, whereas participants in the second group receive BM-MSC intravenous and intranasal (lower 1/3 of nasal passages) route.	Clinical trial active, no results are available yet
NCT02959294	Use of Adipose-Derived Cellular Stromal Vascular Fraction (AD-cSVF) Parenterally in Post-Concussion Injuries and Traumatic Brain Injuries (TBI)	Examine safety and efficacy of parenteral introduction of AD-cSVF, and categorically examine the outcomes according to the elapsed time from original concussive event	1/2	200	16 to 70 years	Patient with concussion syndrome and TBI	Treatment often involves monitoring, physical rest, limiting cognitive activities, and intravenous administration of AD-cSVF	Clinical trial active, no results are available yet
NCT04063215	A Clinical Trial to Determine the Safety and Efficacy of Hope Biosciences Autologous Mesenchymal Stem Cell Therapy for the Treatment of Traumatic Brain Injury and Hypoxic-Ischemic Encephalopathy	Determine the safety and treatment effects of HB-adMSC infusion on global gray and/or white matter, and the structural integrity of grey matter and white matter regions of interest in the corpus callous and corticospinal tracts, as measured by fractional anisotropy and mean diffusivity in specific regions known to correlate with specific neurocognitive deficits in patients after neurological injury	1/2	24	18 to 55 years	Patients with sub-acute or chronic neurological injury (among them TBI)	Patients are infused with 2 × 10^8^ of HB-adMSCs three times over a six week period, spaced 14 days apart	Clinical trial active, no results are available yet

**Table 2:** Clinical trials of non-autologous SC therapies recorded on clinicaltrial.gov.

NCT Number/ References	Official Title	Aim	Phase	Enrolment	Ages	Condition	Treatment	Conclusions
NCT02416492	A Double-Blind, Controlled Phase 2 Study of the Safety and Efficacy of Modified Stem Cells (SB623) in Patients With Chronic Motor Deficit From Traumatic Brain Injury (TBI)	Evaluate the effectiveness, safety, and tolerability of the stereotactic intracranial implantation of allogeneic SB623 cells	2	61	18 to 75 years	Patients with stable, chronic motor deficits secondary to focal TBI with more of 12 months of evolution	The participants were divided into four groups. One group did not receive surgical intervention (control group) and three groups comprised patients who received 2.5 × 10^6^, 5 × 10^6^, or 10 × 10^6^ SB623 cells. The SB623 cells were implanted in the peri-infarct area using stereotactic surgery	Finished, results pending Quality Control (QC) review
NCT02742857	Non-randomized, Open-labeled, Interventional, Single Group, Proof of Concept Study With Multimodality Approach in Cases of Brain Death Due to Traumatic Brain Injury Having Diffuse Axonal Injury	Study the possibility of reversal of brain death induced by TBI with DAI	1	20	15 to 65 years	Patients with brain death due to TBI having DAI	Different therapeutic interventions, two biological (BQ-A Peptide Extract and MSC therapy, MSC transplantation, infusion of intra-thecal bioactive peptides) and two via a device (transcranial laser therapy and median nerve stimulation)	Clinical trial active, no results are available yet

## Data Availability

In clinicaltrial.gov (accessed on 15 October 2020) we carried out the research using the terms “brain injury” and “stem cells” on 15 October 2020. This research led us to find 54 clinical studies. We have considered only clinical trials involving the use of stem cells as a therapeutic treatment for patients with TBI. We excluded clinical studies involving the use of drugs such as erythropoietin that stimulate the production of stem cells or other therapeutic interventions and clinical studies that involved the use of stem cells in diseases other than TBI. Additionally, on PubMed, we looked for studies approved by local ethics committees that evaluated the use of stem cells as a therapeutic treatment in patients with TBI. The keywords used for this research were “head injury”, “stem cells”, “clinical trial”, and “human”.
